# Impact of NAD(P)H: Quinone Oxidoreductase 1 (NQO1) C609T Polymorphism on Lung Cancer Risk

**DOI:** 10.3390/medicina61091504

**Published:** 2025-08-22

**Authors:** Perihan Ozkan Gumuskaya, Kamile Ozkan, Arzu Ay, Tammam Sipahi, Hafize Uzun

**Affiliations:** 1 Department of İnternal Medicine, University of Health Sciences, Prof. Dr. Cemil Tascioglu City Hospital, 34668 Istanbul, Turkey; 2 Department of Biophysics, Medical Faculty of Trakya University, 22030 Edirne, Turkey; kamileozkan@hotmail.com (K.O.); arzuay78@yahoo.com (A.A.); tammam_sipahi@hotmail.com.tr (T.S.); 3 Department of Medical Biochemistry, Faculty of Medicine, Istanbul Atlas University, 34668 Istanbul, Turkey; huzun59@hotmail.com

**Keywords:** lung cancer, NQO1 gene, C > T polymorphism, C609T allele, Turkish patients

## Abstract

*Background and Objectives*: Many studies have demonstrated a relationship between cancer and the NAD(P)H quinone oxidoreductase 1 (NQO1) polymorphism. Lung cancer (LC) is one of the most common malignant diseases and is an expanding global health problem. This study aimed to evaluate the association between the NQO1 C609T polymorphism and LC risk, including its distribution across histopathological subtypes, and to assess its potential as a genetic susceptibility marker. *Materials and Methods*: A prospective study was conducted on 75 LC patients and 65 healthy controls. In this study, the C > T polymorphism occurring at position 609 in the NQO1 gene was examined in Turkish patients with LC. Demographic data and laboratory findings were collected from the patients and the hospital laboratory system. *Results*: The genotype frequencies (CC, CT, and TT) in LC patients were 66.7%, 32.0%, and 1.3%, respectively, compared with 60.0%, 35.4%, and 4.6% in the control group. Chi-square analysis revealed no significant association between the NQO1 C609T polymorphism and LC risk (*p* = 0.433). No correlation was observed between genotype distribution and histopathological subtypes. All patients had a long history of smoking (mean: 38.45 ± 12.14 years and 1.63 ± 0.64 packs/day). *Conclusions:* This is the first study conducted in Turkish people to determine the relationship between the C > T polymorphism occurring at position 609 in the NQO1 gene and the risk of LC. The patients with LC, regardless of their histopathological type, showed no relationship with the polymorphism in the NQO1 gene. Further high-quality investigations with more detailed environmental exposure information and larger sample sizes are warranted to confirm our findings.

## 1. Introduction

Lung cancer (LC) remains one of the most prevalent and deadly malignant diseases worldwide. According to the International Agency for Research on Cancer (IARC), the global cancer statistics for 2022 estimated approximately 20 million new cancer cases and 9.7 million cancer-related deaths. Alarmingly, one in five individuals will develop a malignant tumor during their lifetime, and nearly one in nine men and one in twelve women will die from cancer-related causes [1].

According to GLOBOCAN 2020 data, LC accounted for 17.6% of all cancer cases in Turkey, with age-standardized incidence rates of 41.7 per 100,000 in men and 8.7 per 100,000 in women [2]. Furthermore, data reported by the World Cancer Research Fund indicate that the age-standardized incidence rate in Turkish men in 2022 was exceptionally high at 68.0 per 100,000, among the highest worldwide. In Turkey, LC continues to rank as the most frequently diagnosed cancer and remains the leading cause of cancer-related mortality. According to data from the Turkish Statistical Institute (TSI), deaths attributed to “larynx and trachea/bronchus/lung neoplasms” accounted for 29.7% of all malignant neoplasm-related deaths in 2021 and 29.4% in 2022. When assessed within the context of all-cause mortality, malignant neoplasms represented 14.0% of total deaths in 2021 and 15.2% in 2022 [2,3,4,5].

LC is broadly classified into two main categories, namely small-cell LC (SCLC) and non-small-cell LC (NSCLC), with the latter accounting for approximately 85% of all cases. NSCLC is further subdivided into adenocarcinoma, squamous-cell carcinoma, and large-cell carcinoma [6]. In 2022, LC was the most frequently diagnosed cancer globally, accounting for one in every eight new cancer cases, and remained the leading cause of cancer-related mortality. Among sex-specific statistics, LC ranked as the most common cancer in men, while breast cancer was most prevalent in women [1]. 

The burden of cancer is projected to rise significantly over the next five decades due to global demographic shifts, particularly population aging and growth [7]. Strategic investment in the prevention of key pathophysiological mechanisms underlying cancer could substantially reduce future cancer incidence and mortality, offering both health and economic benefits worldwide [1]. 

NAD(P)H quinone dehydrogenase 1 (NQO1) is an enzyme involved in cellular detoxification and protection against oxidative stress. NQO1 belongs to the quinone oxidoreductase family and is present in various tissues, including the lungs, thyroid, colon, heart, kidneys, liver, cornea, and lens [8]. NQO1 is a critical antioxidant enzyme that reduces quinones to hydroquinone using NADH or NADPH, thereby preventing the formation of highly reactive and harmful semiquinone radicals [9]. Substrates targeting NQO1 have demonstrated promising anticancer effects in clinical trials [10]. Recent evidence suggests that NQO1 may play roles beyond detoxification, influencing tumor biology and treatment response. Elevated NQO1 expression promotes self-renewal and confers therapeutic resistance in NSCLC cell models, underscoring its potential as both a biomarker and a therapeutic target [11]. Therapeutic strategies aimed at restoring NQO1 activity, particularly in individuals homozygous for the C609T polymorphism, may hold significant potential for cancer prevention and treatment [11,12,13].

This study aimed to investigate the relationship between the NQO1 C609T polymorphism and LC risk and to evaluate the distribution of NQO1 genotypes among histopathological LC subtypes. Additionally, this study aimed to assess whether this polymorphism could serve as a potential genetic marker for LC susceptibility in Turkey. 

## 2. Materials and Methods 

### 2.1. Ethical Approval 

All procedures performed in this study involving human participants followed the ethical standards of the institutional and/or national research committee and the 1964 Helsinki Declaration and its later amendments, or comparable ethical standards. The Trakya University Hospital Ethics Committee approved this study for clinical studies (approval number: 09.082007; date: 12 March 2007). 

### 2.2. Data Collection

This prospective study included 75 patients diagnosed with LC and 65 healthy controls, evaluated during routine outpatient visits at Trakya University Hospital between September 2007 and June 2008. All participants met the study eligibility criteria. 

The inclusion criteria were as follows: (1) provision of informed consent, (2) age between 18 and 79 years, (3) absence of chronic diseases other than LC, (4) histopathological confirmation of cancer diagnosis, and (5) newly diagnosed patients who had not yet received chemotherapy or radiotherapy. 

The exclusion criteria were as follows: (1) refusal to participate, (2) presence of chronic diseases other than LC, (3) history of a genetic disorder affecting the NAD(P)H reductase enzyme, (4) pregnancy or breastfeeding, and (5) advanced organ failure. 

Histopathological data were obtained for each LC patient to support clinical characterization. The C > T polymorphism at position 609 in the NAD(P)H quinone oxidoreductase (NQO1) gene was analyzed in Turkish patients with LC. Demographic characteristics and laboratory findings were collected from patient records and the hospital’s laboratory information system. Genotyping of the NQO1 C609T polymorphism was performed using the restriction fragment length polymorphism (RFLP) method. For this purpose, 2 mL of peripheral blood was collected from each of the 75 LC patients and 65 healthy controls. Genomic DNA was isolated using a commercial isolation kit and the salt precipitation method and stored at −20 °C. DNA quality was assessed via electrophoresis on a 0.8% agarose gel. Polymerase chain reaction (PCR) was used to amplify a 212-base pair (bp) region encompassing the polymorphic site at position 609 of the NQO1 gene, using specific primers listed in Table 1.

Agarose gels were prepared at different concentrations according to the type of analysis: 0.8% for DNA quality assessment, 2% for PCR products, and 3% for restriction enzyme digestion products. The gels were prepared in 30 mL of 0.5× TEB buffer and supplemented with 10% ethidium bromide before being poured into casting trays fitted with 13-tooth combs. Electrophoresis was performed in 0.5× TEB buffer at 110 V for 30 min, after which the bands were visualized under UV illumination. For PCR product analysis, 10 μL of each sample was loaded per well. For restriction digestion products, 13 μL of each sample was loaded. The C > T polymorphism at position 609 of the NQO1 gene was detected by digesting PCR amplicons with the HinfI restriction enzyme at 37 °C for 3 h, followed by electrophoretic separation and UV visualization. 

### 2.3. Statistical Analysis

The data was analyzed using SPSS version 21. Categorical variables, including clinical and demographic parameters as well as genotype distributions, were compared between the patient and control groups using the chi-square test. A *p*-value of less than 0.05 was considered statistically significant. Post hoc power analysis was performed to assess this study’s ability to detect differences in genotype distributions. With a total sample size of 140 participants (75 LC patients and 65 healthy controls) and assuming a medium effect size (Cohen’s w = 0.3), the estimated statistical power of this study was approximately 89.9% at an alpha level of 0.05. This indicates limited power to identify smaller effect sizes, which should be considered when interpreting the results. Larger studies are recommended to achieve sufficient statistical power for detecting modest genetic associations. 

## 3. Results 

The mean ages of the 75 LC patients and 65 healthy controls included in this study were 62.69 ± 9.36 and 52.72 ± 16.26, respectively. The patient group consisted of 72 males and 3 females; the control group consisted of 61 males and 4 females. No significant result was found between the patient and control groups in terms of gender in the chi-square test (*p* = 0.704). The small number of females in the patient and control groups effectively prevented obtaining significant results in terms of gender. The characteristics of the patient and control groups are shown in Table 2.

**Figure 1:**
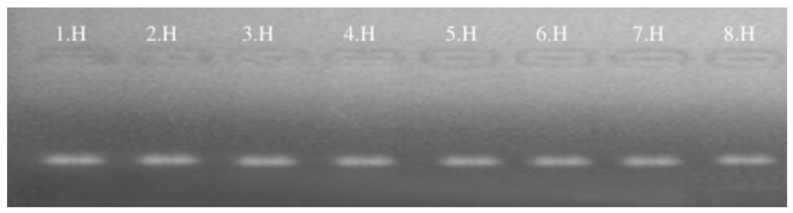
Appearance of DNA under UV light.

The DNAs that were run on a 0.8% agarose gel after isolation and visualized under UV light are shown in Figure 1. The distribution of genotypes after enzyme-cutting procedures in the patient and control groups was examined (Table 3).

The frequencies and percentages of NQO1 genotypes and histopathological types were examined to determine whether there was a relationship between the genotypes of the patient groups and the type of LC (Table 4). In total, 9 patients had adenocarcinoma (12.0%), 19 patients had squamous-cell carcinoma (25.3%), 14 patients had small-cell carcinoma (18.7%), 27 patients had non-small-cell carcinoma (18.7%), and 6 (8.0%) patients had unknown histopathology.

**Figure 2:**
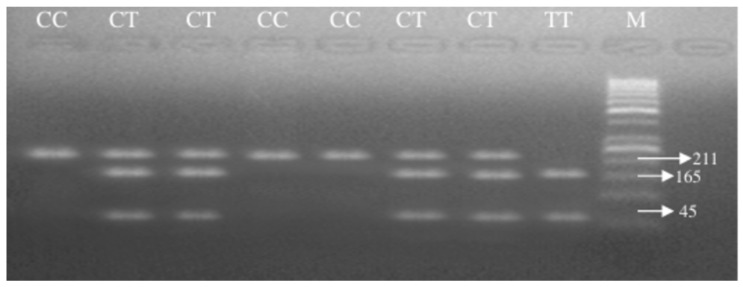
Hinf1 enzyme-cutting result.

**Figure 3:**
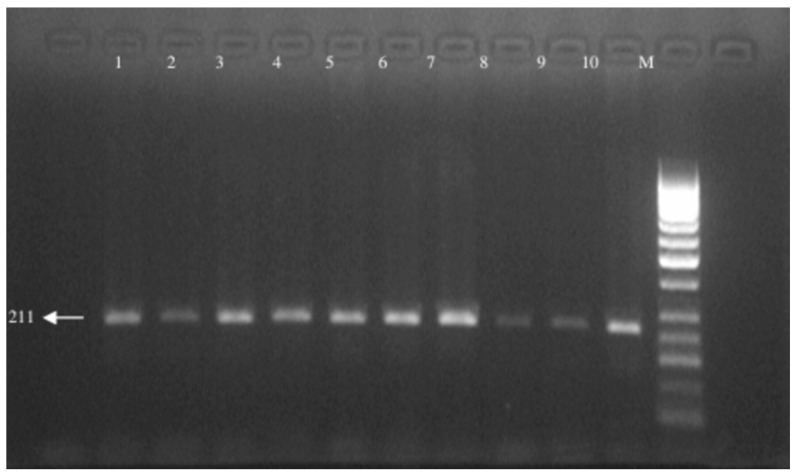
PCR image of the NQO1 gene.

The C > T base change seen in the 609th base pair of the NQO1 gene created an enzyme-cutting site. No cutting was seen in the normal type, and a single fragment was formed in the 211 bp region (CC). Cutting occurred in two regions in the homozygous variant type, and two fragments of 165 and 45 bp were observed (TT). Both alleles were seen in the heterozygous variant type, and three fragments were observed in the 211, 165, and 45 bp regions (CT). The enzyme-cutting results are shown in Figure 2. The PCR image of the NQO1 gene is seen in Figure 3. 

When the participants’ smoking status was examined, all participants had a history of smoking. According to the data obtained, the average smoking duration was 38.45 ± 12.14 years, with an average of 1.63 ± 0.64 packs of cigarettes per day (mean pack-years ≈ 62.7). 

The NQO1 genotype distribution was also examined with the chi-square test. The distribution of CC, CT, and TT genotypes in the patient group was 66.7, 32.0, and 1.3%, respectively, and the distribution in the control group was 60.0, 35.4, and 4.6%, respectively. No significant difference was observed between the patient and control groups in the distribution of the CC, CT, or TT genotypes (*p* = 0.433) (Table 3). There was no significant association between the NQO1 C609T genotype and age at diagnosis. The NQO1 genotype distribution did not significantly differ according to the smoking duration or intensity among cases. No significant association was observed between the NQO1 C609T genotype and histological subtype. 

In our study, no association was found between variant genotypes and LC risk. 

## 4. Discussion 

NQO1 has been reported to participate in the bioactivation of certain environmental procarcinogens found in tobacco smoke and various foods, including nitroaromatic compounds and heterocyclic amines [14]. Numerous investigations have explored the association between NQO1 genetic variants and LC susceptibility; however, the findings remain inconclusive [15]. This study found no significant association between the NQO1 C609T polymorphism and LC risk in the Turkish population. Although the TT genotype was slightly more frequent in the control group (4.6%) than in the patient group (1.3%), the overall genotype distribution (CC, CT, and TT) did not differ significantly between groups. Likewise, no relationship was observed between genotype and the histopathological subtype of LC. All cases occurred in long-term smokers, reinforcing the well-established link between smoking and LC. 

In our study, the distribution of histopathological subtypes among LC patients was as follows: adenocarcinoma (14.0%), squamous-cell carcinoma (26.0%), small-cell carcinoma (14.0%), non-small-cell carcinoma (38.0%), and unknown subtype (8.0%). The genotype frequencies of NQO1 C609T (CC, CT, and TT) did not show significant variation across these subtypes. This lack of association suggests that the NQO1 C609T polymorphism may not differentially influence susceptibility to specific histopathological forms of LC within the Turkish population studied. However, given the relatively small sample size for each subtype, especially for rarer forms, these findings should be interpreted cautiously. Larger studies with more balanced subtype representation are warranted to more definitively assess potential genotype–phenotype correlations. Globally, LC remains the leading cause of cancer-related mortality and has been among the most diagnosed cancers for decades [16]. Genetic polymorphism studies enable the identification of individuals at risk and the discovery of novel therapeutic targets. The NQO1 C609T variant, a C→T substitution in exon 6 resulting in a serine-for-proline substitution, has been associated with altered enzyme activity. This functional change can influence carcinogen detoxification and potentially contribute to cancer susceptibility.

LC remains the leading cause of cancer death. Globally, LC has been the most commonly diagnosed cancer for the last several decades [16]. Genetic polymorphism studies provide the opportunity to identify individuals at risk for disease and to identify new targets for drug therapy. The genetic polymorphism in NQO1 is a C-T substitution in the 609th location in exon 6, resulting in a serine substitution for proline in the protein. Homozygous C→T conformers in the 609th location are responsible for the disruption of NQO1 fragments. In this study, we investigated the presence of polymorphisms in the NQO1 gene in Turkish patients diagnosed with LC, using the PCR-RFLP method for detection. Our results did not detect any significant NQO1 gene polymorphism among the patients analyzed. To our knowledge, this is the first study to examine the NQO1 polymorphism in a Turkish LC population. Our findings are noteworthy because NQO1 polymorphisms, particularly the C609T variant, have previously been associated with increased susceptibility to LC in some populations. Huang et al. [17] observed no significant overall effect in their larger meta-analysis, including 37 studies, 8493 patients, and 10,999 controls, though a modest association was noted in the small-cell LC subgroup. They stated that the T allele may be associated with risk in small-cell LC, but it did not show a strong effect in histology subgroups, especially in Whites or Asians. In another meta-analysis, Liu et al. [15] suggested that the NQO1 609T allele is a low-penetrant risk factor for developing LC in Chinese individuals. However, this commonly studied polymorphism was not observed in the Turkish population examined in our study. The absence of such polymorphisms in our cohort may reflect ethnic or regional genetic variation, environmental influences, or population-specific carcinogenic exposures. Chao et al.’s meta-analysis of NQO1 C609T and LC risk [18] reported no clear link among White, Asian, or Black people. Also, the Turkish results align with a prior Turkish hepatocellular carcinoma study that found no association between NQO1 C609T and cancer risk, reinforcing the notion of population-specific genetic backgrounds affecting polymorphism prevalence [19]. In an older study in Taiwan, Lin et al. [20] showed that, similar to our study, the NQO1 (Pro187Ser) polymorphism was not strongly associated with LC risk in the general population; however, in individuals who smoked, the normal (wild-type) form of NQO1 increased the risk of adenocarcinoma by approximately 2.5-fold.

All cases in our series had a substantial history of cigarette smoking (mean pack-years ≈ 62.7). Because smoking is a strong risk factor for LC, the high and relatively homogeneous smoking exposure in our case group may have obscured small/moderate effects of the NQO1 C609T polymorphism. This limits our ability to disentangle the independent effect of the genotype from the dominant effect of smoking in this cohort; we have added this as an explicit limitation of this study. Masroor et al. [21] reported a significant association between the NQO1 C609T polymorphism and increased risk of NSCLC in an Indian population, where a substantial proportion of patients are non-smokers exposed to biomass fuels and other environmental carcinogens. Their findings suggest that this genetic variant may contribute to LC susceptibility in certain ethnic groups with distinct environmental exposures. However, in contrast, our study conducted in a predominantly smoking Turkish population did not find a significant relationship between the NQO1 C609T polymorphism and LC risk, irrespective of histopathological subtype. This discrepancy likely reflects differences in allele frequencies, environmental factors, and gene–environment interactions across populations. These results highlight the importance of considering both genetic and environmental contexts and underscore the need for population-specific studies to elucidate the role of genetic variants, such as NQO1 C609T, in cancer risk. 

Banerjee [22] found a significant association between the NQO1 C609T polymorphism and increased LC risk among male smokers in Eastern India, suggesting a gene–environment interaction specific to tobacco exposure. In contrast, our study did not reveal such an association in the Turkish population, highlighting the importance of ethnic and environmental context when evaluating genetic risk factors for LC. 

Our findings highlight the importance of population-specific genetic studies in cancer research. While NQO1 polymorphisms have been implicated in other ethnic groups, their role in the Turkish population appears to be limited or absent, at least within the sample size we evaluated. This result also raises the possibility that alternative molecular mechanisms or other genetic polymorphisms may play a more prominent role in LC susceptibility among Turkish individuals. Furthermore, the differences in findings across populations underscore the need for larger multi-centered studies involving Turkish subpopulations and possibly matched healthy controls. Such studies may clarify whether the absence of NQO1 polymorphisms in this study is representative of the general Turkish population or specific to the cohort analyzed.

Previous studies conducted in Turkish populations have investigated the role of the NQO1 C609T polymorphism in various cancers. Ergen et al. [23] reported a potential association between this polymorphism and prostate cancer, suggesting its role may be cancer-type specific. In contrast, Akkiz et al. [19] found no association between the NQO1 C609T variant and hepatocellular carcinoma risk in Turkish individuals, while Sirma et al. [24] similarly observed no significant relationship in pediatric de novo acute leukemia. Consistent with these findings, our study further supports the notion that the NQO1 C609T polymorphism does not significantly contribute to cancer susceptibility across multiple tumor types in the Turkish population, including LC. 

Recent studies continue to explore the complex role of NQO1 in cancer pathogenesis and its clinical implications. Ghorbani et al. [25] reviewed the significance of NQO1 polymorphisms in the prevention, diagnosis, and treatment of gastrointestinal cancers, emphasizing its potential as a biomarker and therapeutic target in specific tumor types. Similarly, Ichikawa et al. [26] demonstrated that NQO1 expression in non-neoplastic esophageal squamous epithelium carries prognostic value for patients with esophageal cancer, further supporting its involvement in cancer biology beyond tumor tissues. In contrast with these findings, our study did not find a significant association between the NQO1 C609T polymorphism and LC risk in the Turkish population. This discrepancy may reflect the tissue-specific roles of NQO1 or population-based genetic variability. These differences highlight the importance of conducting cancer-type and population-specific genetic studies to accurately assess the clinical utility of NQO1 as a biomarker or therapeutic target. 

Regarding the potential association between the NQO1 C609T polymorphism and chronic obstructive pulmonary disease (COPD), none of the patients included in our study had a COPD diagnosis. Therefore, we were unable to evaluate any relationship between this polymorphism and COPD presence or severity. Given the well-established link between smoking, COPD, and LC, future studies including patients with coexisting COPD may provide valuable insights into shared pathophysiological mechanisms and the role of NQO1 genetic variants in these overlapping conditions. 

Although this study focused on the NQO1 C609T polymorphism, LC susceptibility is known to result from complex interactions between multiple genetic variants and environmental exposures [27,28]. For example, exposure to biomass smoke has been implicated as a significant risk factor in certain populations, and polymorphisms in detoxification genes, such as the GSTM1 and CYP450 families, have been shown to modulate LC risk via gene–environment interactions [29]. Persons with both GSTM1 deletion and elevated PAH-DNA adducts may represent a sensitive subpopulation concerning carcinogens in tobacco smoke and other environmental media [30]. Future studies incorporating a wider array of genetic markers, along with detailed environmental exposure data, are essential to better elucidate these multifactorial influences. 

This study has some limitations. The relatively small sample size may have limited the detection of modest genotype effects. Additionally, since all participants were smokers, we could not assess gene–environment interactions between NQO1 polymorphisms and smoking. The uniform heavy smoking exposure may have masked potential associations. Other environmental factors and comorbidities were not evaluated. Finally, as a single-center study in a Turkish population, the results may not be generalized to other groups. Larger studies with more diverse populations are needed to confirm our findings. 

Our study provides preliminary insights into the association between NQO1 C609T polymorphisms and LC risk, highlighting the limited clinical utility of routine genotyping for risk stratification in the Turkish population. The application of the PCR-RFLP method, a well-established and reliable genotyping technique, supports the robustness of our findings. Nevertheless, the relatively small sample size constitutes a limitation. Therefore, larger-scale studies encompassing diverse cohorts from multiple regions and ethnic backgrounds within Turkey are warranted to validate and extend these results. In conclusion, while the NQO1 C609T polymorphism does not appear to be a major biomarker for LC susceptibility among Turkish individuals, our research provides an important foundation for future investigations into the complex interplay between genetic and environmental factors in LC pathogenesis. Future, larger studies should include non-smoking cases and detailed environmental exposure data to permit formal gene–environment interaction testing. 

## Figures and Tables

**Table 1:** The primers used in this study.

**F: 5′-TCC TCA GAG TGG CAT TCT GC -3′**
**R: 5′- TCT CCT CAT CCT GTA CCT CT -3′**

**Table 2:** Characteristics of patient and control groups.

	Patients	Controls
***Gender***		
**Female, n (%)**	3 (4.0%)	4 (6.2%)
**Male, n (%)**	72 (96.0%)	61 (93.8%)
**Smoking**	75 (100%)	65 (100%)
***Histopathology***		
**Adenocarcinoma**	9 (12.0%)	
**Squamous-cell carcinoma**	19 (25.3%)	
**Small-cell carcinoma**	14 (18.7%)	
**Non-small-cell carcinoma**	27 (36.0%)	
**Unknown**	6 (8.0%)	

**Table 3:** Distribution of NQO1 genotypes in patient and control groups.

Genotype	Patient	Control	*p*-Value
**CC**	50 (66.7%)	39 (60.0%)	
**CT**	24 (32.0%)	23 (35.4%)	0.433
**TT**	1 (1.3%)	3 (4.6%)	

**Table 4:** Distribution of NQO1 genotypes according to histopathological types.

Histopathology	CC	CT	TT
**Adenocarcinoma**	7 (14.0%)	2 (8.3%)	
**Squamous-cell carcinoma**	13 (26.0%)	6 (25.0%)	
**Small-cell carcinoma**	7 (14.0%)	7 (29.2%)	
**Non-small-cell carcinoma**	19 (38.0%)	7 (29.2%)	1 (100%)
**Unknown**	4 (8%)	2 (8.3%)	

## Data Availability

The original contributions presented in this study are included in this article. Further inquiries can be directed to the corresponding author(s).
